# Production loss among employees perceiving work environment problems

**DOI:** 10.1007/s00420-014-1003-0

**Published:** 2014-11-29

**Authors:** Malin Lohela-Karlsson, Jan Hagberg, Gunnar Bergström

**Affiliations:** Karolinska Institutet, Stockholm, Sweden

**Keywords:** Work conditions, Work performance, Psychosocial work environment, Productivity loss

## Abstract

**Objectives:**

The overall aim of this explorative study was to investigate the relationship between factors in the psychosocial work environment and work environment-related production loss.

**Methods:**

Employees at a Swedish university were invited to answer a workplace questionnaire and were selected for this study if they reported having experienced work environment-related problems in the past 7 days (*n* = 302). A stepwise logistic regression and a modified Poisson regression were used to identify psychosocial work factors associated with work environment-related production loss as well as to identify at what level those factors are associated with production loss.

**Results:**

Employees who reported having experienced work environment problems but also fair leadership, good social climate, role clarity and control of decision had significantly lower levels of production loss, whereas employees who reported inequality and high decision demands reported significantly higher levels of production loss. Never or seldom experiencing fair leadership, role clarity, equality, decision demands and good social climate increase the risk of production loss due to work environment problems, compared to those who experience these circumstances frequently, always or most of the time.

**Conclusions:**

Several psychosocial work factors are identified as factors associated with a reduced risk of production losses among employees despite the nature of the work environment problem. Knowledge of these factors may be important not only to reduce employee ill-health and the corresponding health-related production loss, but also reduce immediate production loss due to work environment-related problems.

## Introduction

Every year, a significant proportion of employees in various occupational groups (Dew et al. 2005) come to work despite illness (Hansen and Andersen 2008). The prevalence of this problem has been reported to be 30–73 % (Hansen and Andersen 2008; Aronsson et al. 2000; Aronsson and Gustafsson 2005; Robertson et al. 2012). In another study, about one-fifth of the employees reported health-related problems in the past 7 days (Karlsson et al. 2013), with a majority reporting that these problems affected their ability to perform at work. Years of research have shown that employee health is affected by the work environment. Despite our increased knowledge about work environment hazards, companies still frequently struggle with poor working conditions. The consequences show up later, not only in terms of ill-health. Employees experiencing work environment problems can also be affected directly in terms of decreased ability or desire to work, resulting in production loss to the company—as a result, for example, of work-related stress (Callen et al. 2013), poor workplace management (Wynne-Jones et al. 2011), poor workplace culture (Wynne-Jones et al. 2011), high job demands (Johns 2010) and low levels of job control (van den Berg et al. 2011; Lerner et al. 2010).

Production loss is a measure that often combines absenteeism with presenteeism. Presenteeism can be defined as reduced performance or production at work due to an employee’s impaired health or to a particular health condition (Brooks et al. 2010). Production loss is a frequent consequence of presenteeism and can be defined as the difference between an employee’s normal performance and his or her performance while affected by the problem—that is, if for example, a health-related problem reduces the employee’s performance to a lower level, a production loss will arise (Karlsson et al. 2013). The cost of presenteeism and the corresponding production loss have been given several estimates, which suggest that 77 % of total lost production at the workplace is related to this problem (Stewart et al. 2003). Research today has so far mostly evaluated health-related production loss. However, poor working conditions can also result in reduced performance among employees affected by problems in the work environment.

A recent published study (Karlsson et al. 2013) showed that production loss due to work environment-related problems exists, and that the average reduction due to this problem is higher than the reduction that is due to health-related problems. This indicates that employers have at least two potential costs due to work environment problems: immediate costs in terms of work environment-related production loss and costs due to health-related production loss, which can occur both immediately and in the future. Production loss due to health-related problems has drawn a lot of public attention. However, production loss directly attributed to work environment-related problems has rarely been investigated (Karlsson et al. 2013).

If production loss due to problems in the work environment exists at a company, there is a potential to decrease the related costs by improving the work environment or reducing the problems perceived by the employees. Reduced production loss will most likely improve company productivity, as employees’ output will improve in relation to the time they put in.

Work environment problems can be concerned with different parts of the work environment and of both physical and psychosocial characteristics. While some factors in the work environment may cause a perceived problem, others could increase or decrease the outcome of the problem. It is possible that some factors in the work environment are more significant than others as to how they affect employees’ perception of problems in terms of their ability to perform. Some factors may cause production loss to a higher degree, whereas others may reduce production loss. It is important that both types of work environment factors be identified. Another question is whether these factors always reduce production loss, or whether they need to be perceived at a specific level to function in that way—that is, is it sufficient to improve only this or that specific factor, or must it be improved to a certain level in order to reduce production loss? In this study, we are particularly interested in factors related to the psychosocial work environment and how they impact the performance of employees experiencing work environment problems.

Work environment problems have previously been studied in relation to ill-health and to some extent as a determinant of health-related production loss. They have been studied to a lesser extent; however, in relation to their effect on employees’ ability to perform at work. Work environment problems appear to be associated with production loss when perceived by employees as problems (Karlsson et al. 2013). This indicates that work environment problems affect employees’ ability to perform to a significant extent and that employers have a lot to gain if they can reduce work environment problems, potentially ensuring less production loss and fewer health-related problems immediately and in the future. The reasons why perceived work environment problems affect employees’ ability to perform and what the factors are that do affect their ability to perform at work remain unexplored. This study adds to existing knowledge by investigating what psychosocial work factors are related to work environment-related production loss. To further add to this knowledge, different levels of psychosocial work factors are investigated in order to determine whether some levels correlate with more or less work environment-related production loss.

## Aim

The overall aim of this explorative study was to investigate the relationship between factors in the psychosocial work environment and work environment-related production loss. More specifically, the aim was to answer these two research questions:

1. What psychosocial work factors are associated with work environment-related production loss?
2. What level of those psychosocial work factors is associated with the work environment-related production loss?

## Methods

A multilevel intervention was performed between 2007 and 2011 at a Swedish University. The intervention used, which was based on the AHA method (Bergström et al. 2008), incorporates both an individual and a group level perspective. The method is based on three main steps: screening, feedback and intervention and uses evidence-based methods to improve psychosocial work conditions, reduce work-related ill-health and improve company productivity. An extensive description of the main study is reported elsewhere (Bergström et al. 2008; Bergström et al. 2004; Roos et al. 2005).

A modified version of AHA was adapted to this setting and had a special focus on the psychosocial work environment. The screening, which was distributed to all employees, was conducted every second year starting in 2007, by means of a validated questionnaire. The last screening, performed in 2011, also included questions related to employee health and production loss. The results were fed back on both the organizational and the individual level. Results from the screening were fed back to the groups with the aim of discussing the results and creating action plans for areas in need of improvement and for areas that were satisfactory and that should be maintained.

### Ethical approval

The study was approved by the Ethical Committee of Karolinska Institutet (AHA; Dnr 00-012 and Dnr 2010/1516-32).

### Material and data collection

This cross-sectional study includes all employees who had been employed at the university at least part-time and for at least 6 months and who answered the questionnaire in 2011. The invitation was distributed to all study participants by e-mail and was followed up with two reminders. The responses were anonymous to employers and were forwarded to the research team. Participation was encouraged but voluntary, and a written informed consent was obtained from the employees.

All participants were asked if they had experienced work environment-related problems the past 7 days. Work environment-related problems were defined as any physical, psychological or social problems that might arise in the work environment. Only those that answered yes were asked to answer a following question about production loss related to problem. Those answering yes were included in the further analyses. A total of 3,515 employees responded to the work environment survey in year 2011 (68 % response rate). A comparison of participants and non-participants revealed certain differences: Both the proportion of women was smaller (59 vs. 66 %; *p* < 0.001), and the proportion of researchers was smaller (58 vs. 73 %; *p* < 0.001) among the participants than among the non-participants. Of those, 739 employees reported that they had experienced work environment problems the past 7 days. More than half of these also reported health-related problems in the same period. The population in this study consists of the employees who had only reported work environment problems in the past 7 days (*n* = 307). The decision to define the study population in this way was based upon a previous study showing that employees experiencing both types of problems are a special group that needs to be studied independently (Karlsson et al. 2013). This group reported higher levels of production loss than did the group that only experienced work environment-related problems. The previous study also showed that in the group with a combination of problems, production loss was to some extent associated with a different set of factors than in the group with only one problem.

### Measurements

*Work environment-related production loss* was collected using the question: During the past 7 days, how much did your work environment-related problems affect your performance while you were working? Think about days you were limited in the amount or kind of work you could do, days you accomplished less than you would like or days you could not do your work as carefully as usual. If work environment-related problems affected your work only a little, choose a low number. Choose a high number if work environment-related problems affected your work a great deal. The scale ranged from 0 to 10 where 0 = Work environment problems had no effect on my work and 10 = Work environment problems completely prevented me from working. This scale was converted from 0–10 to 0–100 to capture the percentage loss of work time. The question has shown to be valid (Karlsson et al. 2013).

The *psychosocial work* *factors* included are fair and empowering leadership, social climate and innovative climate, control of work pacing, control of decisions, quantitative demands, decision demands, social support from colleagues and from one’s manager, role clarity, work-life balance, work-related challenges, feedback, goal clarity and inequality. These were measured using the validated QPS Nordic questionnaire (Dallner et al. 2000). The validated scale developing leadership (Larsson 2003) and co-workership (Tengblad 2010) were also included. The responses were scored using a 5-point Likert scale with response categories ranging from, for example, “very seldom or never” to “very often or always.” Items in each of the indices were summed up and standardized to generate a total score ranging from 1 to 5. A high score is favorable in all of the indices except inequality. In that case, a lower score indicates less inequality, which is considered better.

### Statistical analyses

In order to answer the research questions, the analysis was performed in several steps and used an explorative approach. In the first step, the correlations between work environment-related production loss and psychosocial work factors were tested for using Spearman’s rank correlation coefficient. That test was chosen due to the scale of the variables in the test, where the independent variables are ordinal numeric, and the dependent variable is continuous. Nonsignificant variables (*p* value >0.20) were excluded from further analysis.

To identify the psychosocial work factors of importance for work environment-related production loss, a stepwise logistic regression was used. This type of regression was chosen as the linear relationship between the dependent and the independent variables was weak, and we were unable to identify an acceptable non-linear model. The dependent variable was categorized in different levels; ≥30, ≥40, ≥50, ≥60, and stepwise regression analyses were performed for each level. These levels were chosen from the distribution of the production loss levels among the study population; that is, few employees had scored less than 30 or higher than 60 on the scale. In this step, the independent factors were used as continuous variables. The analyses were performed using both a forward and backward approach to confirm the final model. A variable was entered into the model if the probability of its score statistic was <0.05 and was removed if the probability was >0.10. The final results of the stepwise procedure were then included in a modified Poisson regression (Zou 2004) to identify the risk ratio (RR) of each psychosocial work factor. A 95 % confidence interval was chosen as inclusion criteria. This was done because risk ratios are easier to interpret than are odds ratios, which are presented in the logistic regression. In a final step, we also tested alternative models and compared the AIC values (Akaike’s Information Criterion).

In order to answer the second research question, the independent variables identified in the previous step described above were categorized into quartiles. This was done to determine whether different levels of the independent variables were related to increased or decreased levels of production loss. The highest quartile for each variable was chosen as the reference category, that is, those perceiving the work environment factors to be very good. A modified Poisson regression was then performed to identify levels that increased or decreased production loss. The same procedure was performed for each level of production loss (≥30, ≥40, ≥50, ≥60). All the analyses were performed with SPSS version 22.

## Results

Three hundred and seven employees reported that they had work environment problems only. A majority of this group was employed as researchers, was female, and had been working for more than 2 years at the workplace (Table 1). This group does not significantly differ from the total population except in that it has a higher proportion of women (72 vs. 66 %). Eighty-nine percent of the employees reported that the perceived work environment problems affected their performance at work—that is, caused work environment-related production loss.

**Table 1:** Background information of all responders to the survey as well as the study population; that is, employees experiencing work environment problems only

	Total population (*n* = 3,515)	Work environment problems (*n* = 307)	*p* value
Occupation [*n* (%)]			0.426^a^
Researcher	2,022 (58)	170 (55)	
Administrative staff	1,493 (42)	137 (45)	
Manager [*n* (%)]			0.466^a^
Yes	655 (19)	62 (20)	
No	2,860 (81)	245 (80)	
Gender [*n* (%)]			0.030^a^
Men	1,190 (34)	87 (28)	
Women	2,325 (66)	220 (72)	
Age [*n* (%)]			0.513^b^
−29	463 (13)	41 (13)	
30–39	1,096 (31)	98 (32)	
40–49	796 (23)	71 (23)	
50–59	710 (20)	65 (21)	
60+	450 (13)	32 (11)	
Years at current workplace [*n* (%)]			0.380^b^
<1 year	270 (8)	22 (7)	
1–2 years	709 (20)	68 (22)	
3–5 years	954 (27)	85 (28)	
6–10 years	582 (17)	53 (17)	
>10 years	1,000 (28)	79 (26)	
Production loss due to work environment problems [*n* (%)]			
Yes	–	274 (89)	
No	–	28 (9)	
Missing	–	5 (2)	

^a^Tested with uncertainty coefficient

^b^Tested with Kendall’s tau-b

### Psychosocial work factors associated with production loss

Five individuals had internal missing on production loss and were excluded from the analyses. Of the described psychosocial work factors, a total of seven factors were associated with different levels of the work environment-related production loss (Table 2). The estimates shown in Table 2 are adjusted for all other presented variables. To facilitate the presentation, results are only given for levels ≥30 and ≥60. Levels ≥40 and ≥50 showed equal patterns as level ≥60, that is, the same variables were associated with these levels as with ≥60 and with similar RR. Fair leadership and role clarity were factors that were included as associated factors in most levels of production loss. Employees experiencing higher levels of fair leadership have a decreased risk of perceiving production loss. Also, for each level of production loss employees who perceive themselves to have more role clarity have a decreased risk of experiencing production loss. Perceiving inequality at the workplace was associated with an increased risk of production loss. A good social climate was associated with decreased levels of production loss (RR 0.866) at the lower levels of production loss (≥30). High decision demands were associated with an increased risk of production loss at the lower levels of production loss (≥30, ≥40). Work control was also associated with production loss. Experiencing high control of decision was associated with a decreased level of production loss. However, this association was only found for the lowest level of production loss. High control of work pacing showed a similar pattern but with an increased risk of production loss.

**Table 2:** Final models of psychosocial work factors affecting different levels of work environment-related production loss (*n* = 302)

Variables	Production loss ≥30^a^		Production loss ≥60^b^	
	RR	CI^c^	RR	CI^c^
Fair leadership			0.787	0.626–0.989
Role clarity	0.850	0.759–0.952	0.712	0.562–0.903
Decision demands	1.236	1.029–1.484		
Inequality	1.177	1.044–1.327	1.272	1.035–1.565
Social climate	0.866	0.759–0.988		
Control of decisions	0.818	0.675–0.991		
Control of work pacing	1.209	1.023–1.428		

^a^AIC 487

^b^AIC 283

^c^95 % CI

### Levels of psychosocial work factors and production loss

The different levels of psychosocial work factors and how they are related to production loss are presented in Table 3. The table presents both the unadjusted RR—that is, the single factors’ association with the work environment-related production loss—and the adjusted RR—that is, the independent contribution of each factor when the other factors in the model were controlled for.

**Table 3:** Levels of psychosocial work factors and how they are related to different levels of production loss (*n* = 302)

Variables	≥30					≥60				
	*N* (*n*)^a^	RR Unadjusted	CI	RR Adjusted	CI	*N* (*n*)^a^	RR Unadjusted	CI	RR Adjusted	CI
Fair leadership										
Poor (1–2.34)						93 (17)	**2.895**	1.527–5.488	1.795	0.915–3.521
Neither nor (2.35–3.34)						74 (16)	1.091	0.465–2.558	0.854	0.382–1.905
Good (3.35–4.01)						62 (15)	1.257	0.577–2.736	1.056	0.509–2.190
Very good (4.02–5.00)						73 (35)	1		1	
Role clarity										
Poor (1–3.01)	78 (41)	**1.987**	1.397–2.825	**1.669**	1.162–2.397	78 (16)	**2.486**	1.329–4.652	1.896	0.972–3.697
Neither nor (3.02–3.68)	83 (53)	1.359	0.899–2.055	1.359	0.894–2.066	83 (16)	1.108	0.503–2.441	0.999	0.451–2.211
Good (3.69–4.34)	64 (48)	1.265	0.846–1.892	1.278	0.871–1.874	64 (18)	0.598	0.244–1.456	0.586	0.245–1.399
Very good (4.35–5.00)	77 (61)	1		1		77 (33)	1		1	
Decision demands										
Low (1–3.01)	62 (38)	1.346	0.917–1.977	**1.470**	1.005–2.148					
Neither nor (3.02–3.68)	106 (71)	1.394	0.946–2.054	**1.445**	1.012–2.063					
Rather high (3.69–4.01)	63 (44)	1.121	0.766–1.641	1.242	0.881–1.751					
High (4.02–5.00)	71 (50)	1		1						
Equality										
Very poor (1–1.01)	82 (45)	**1.642**	1.184–2.276	**1.420**	1.020–1.978	82 (17)	1.922	0.926–3.989	1.673	0.859–3.259
Poor (1.02–1.68)	85 (46)	**1.480**	1.058–2.070	1.369	0.986–1.900	85 (14)	**2.194**	1.093–4.404	**2.092**	1.079–4.055
Neither nor (1.69–2.34)	71 (58)	0.814	0.539–1.230	0.801	0.538–1.195	71 (27)	1.061	0.476–2.364	1.043	0.484–2.244
Good (2.35–5.00)	64 (54)	1		1		64 (25)	1			
Social climate										
Poor (1–2.34)	57 (23)	**2.235**	1.453–3.438	**1.633**	1.016–2.622					
Neither/nor (2.35–3.34)	88 (51)	**1.736**	1.109–2.717	1.558	0.989–2.452					
Good (3.35–4.01)	85 (69)	1.219	0.751–1.980	1.105	0.673–1.814					
Very good (4.02–5.00)	72 (60)	1		1						
Control of decision										
Low (1–2.51)	94 (47)	**1.534**	1.088–2.162	1.329	0.897–1.968					
Neither nor (2.52–3.01)	61 (43)	**1.527**	1.075–2.171	**1.497**	1.046–2.141					
Rather high (3.02–3.71)	69 (51)	1.354	0.925–1.982	1.312	0.901–1.912					
High (3.72–5.00)	78 (62)	1		1						
Control of work pacing										
Low (1–3.26)	81 (59)	0.931	0.678–1.278	1.415	0.996–2.012					
Neither nor (3.27–3.76)	58 (41)	0.906	0.649–1.264	1.229	0.866–1.744					
Rather high (3.77–4.26)	76 (46)	0.943	0.662–1.342	1.043	0.744–1.461					
High (4.27–5.00)	87 (57)	1		1						

^a^ *N* number of total cases in the defined category, *n* number of exposed cases in the category, i.e., number of employees reporting a production loss ≥30/≥60

The result shows that the RR is significantly higher for the worst level of work environment factors compared to the RR for those who perceived their work environment to be very good. Perceiving the work environment as something in between, neither extremely good nor extremely bad, was not significantly associated with an increased risk of production loss, except for decision demands and perceived workplace equality. These two factors were associated with an increased risk of production loss, even for the third quartile for the different levels of production loss.

## Discussion

The aim of this study was to investigate the relationship between factors in the psychosocial work environment and work environment-related production loss. We were particularly interested in identifying the psychosocial work factors that were associated with work environment-related production loss and establishing the levels at which these psychosocial work factors were associated with the production loss.

### Psychosocial work factors impacting on work environment-related production loss

Role clarity was associated with all investigated levels of production loss (≥30, ≥40, ≥50, ≥60). Fair leadership and equality was associated with almost all levels and seem to be more important than the other factors included in the model. A fair leader and good role clarity seem to reduce the level of production loss. Previous literature referred to workplace resources that could help people manage specific work situations. High job control, for example, has been seen as a resource to handle high job demands (Bakker and Demerouti 2007). In this study, a perception of fair leadership and high role clarity seem to act as workplace resources that help workers who experience work environment problems to manage the impact of these problems on their performance at work. In another study, it was hypothesized that job control would reduce the perception of job demands, and that supervisor support would result in fewer job demands (Luchman and González-Morales 2013). This explanation would also be valid in terms of the results of this study. Workers who perceive fair leadership and role clarity are less affected by their work environment problems and do not perceive them to be as onerous as do others who lack these resources (Berg et al. 2011).

Perceiving equality is associated with a decrease in production loss at several levels. A previous study showed that workplaces with gender equality had lower levels of psychological distress among the employees than did gender unequal workplaces (Elwér et al. 2013). The pattern is the same as that shown in this study; that is, equality in the workplace is associated with fewer negative consequences. However, as we have no information about the cause of the work environment problem, it is possible that inequality is the cause of the problem, and that equality is a factor that minimizes the outcome of the problem. Besides the factors previously mentioned, social climate, job demands and job control were factors that were found to be important in this study. Job control has also been shown to be important for reducing production loss among workers with reduced work ability (Berg et al. 2011). However, the factors identified in this study were only significant for the lower levels, indicating that they might act as so-called buffering factors, i.e., reduce the level of production loss at lower levels of production loss. Several of these factors have been found to be important for the level of health-related production loss as well (Lack 2011).

The knowledge about what psychosocial work factors are associated with production loss as well as how different psychosocial work factors impact production loss can be key in the identification of the causes of production loss and the design of interventions to improve the psychosocial work environment. With this in mind, these interventions will not only reduce employee ill-health and the corresponding health-related production loss, but also reduce immediate production loss caused by work environment-related problems.

### Level of psychosocial work factors and production loss

Perceiving the supervisor as unfair in most aspects (level 1–2 on the Likert scale) was related to an increased risk (unadjusted) of production loss compared to perceiving a supervisor as fair in most aspects (level 4–5 on the Likert scale). With regard to role clarity, perceiving role clarity as poor (level 1–3 on the Likert scale) is associated with an increased risk of production loss compared to perceiving role clarity as very good (level 4.35–5 on the Likert scale). The results of this study indicated that never (Likert scale level 1) or seldom (Likert scale level 2), and in some cases sometimes (Likert scale level 3), perceiving fair leadership, role clarity, equality, reasonable decision demands and a good social climate increase the risk of production loss due to work environment problems compared to when these benefits are perceived very often/always or most of the time. This could be interpreted as independent of the work environment problem; these factors might act as buffering factors and could therefore limit the consequences of the problem. This is suggested to be investigated in future studies. The results for work pacing were unstable and contradictory and should be interpreted with caution.

### Methodological considerations

An explorative approach was used in this study to identify psychosocial work factors of relevance for work environment-related production loss. The included factors were limited to psychosocial work factors and did not capture other aspects of the work environment that could be of relevance for work environment-related production loss. One limitation of the study is that no information on the nature of the work environment problem was gathered. This means that the described psychosocial factors could both be part of the problem itself (for instance conflicting roles at work) or modify an independent work environment problem. For instance, a fair and supportive leader could make it possible to maintain production despite different upcoming work environment problems.

Using an explorative approach could affect our ability to generalize the results, as the results could be specific for this particular study population. Several regression analyses were performed, such as stepwise backward and forward logistic regression, combined with the statistical measure Akaike (AIC) obtained from a modified Poisson regression to ensure that the final models were not only identified by chance. However, a replication of the study needs to be done on another population to evaluate whether the identified psychosocial work factors are valid and generalizable. Whether the identified associations are similar, or vary, across subgroups such as occupational category, age, gender, etc., also needs to be investigated.

Another possible limitation of this study is that it included a subsample of the total population. First of all, only 68 % of the employees invited chose to respond to the questionnaire—which could affect the generalizability of the results. The reason for the non-participation of the other employees is unknown. A significance test on the groups showed some differences with regard to the proportion of women as well as the proportion of researchers answering the questionnaire. A significance test was performed on this group and on the total sample to test for differences in several background data. The two groups did not significantly differ except that the proportion of women was higher in the group reporting work environment-related problems only. It is possible that employees among the non-participants also perceived work environment-related problems, and that the lack of this information affects the generalizability of the results.

This study is cross-sectional and consists of self-reported data. The cross-sectional design limits our ability to draw any conclusion about causality. The research questions in this study, which involved investigation of the relationship between psychosocial work factors and production loss using a self-rated measure, require a cross-sectional study design inasmuch as performance and production loss are highly variable entities and are affected by present health and work status. Thus, it is essential to capture present work situation, that is, the perception of the work environment and production loss at the same time point. It is possible that the consequences of a perceived problem will change over time depending on how the person is affected by this problem and that other, or additional, factors in the psychosocial work environment will be found important in a longitudinal study.

It may also be that some part of the associations among the constructs is affected by common method variance. However, since all our measurements were gathered by means of self-report questionnaires, this variance should reasonably be equally distributed among the variables—that is, common method variance may have influenced the strength of the associations, but not the relative impact of the independent variables when compared to one another. Furthermore, there are some indications that the influence of common method variance may be overestimated—for instance, many examples exist in both our material and elsewhere that there are necessarily no associations between variables just because they are measured by self-report instruments in a cross-sectional design (Spector 2006).

Employees’ attitudes toward their job—such as job motivation, job satisfaction and organizational commitment—could be other factors that might affect the levels of production loss. Employees who are highly motivated in their job could, for example, be less affected by the perceived work environment problem and therefore have lower levels of production loss. It is suggested that future studies also include this when evaluating factors that might affect the level of production loss among employees.

### Conclusion and future research

This study evaluates the impact of different psychosocial work factors on work environment-related production loss among employees perceiving work environment problems. Several psychosocial work factors are identified as factors associated with a reduced risk of production loss among the employees despite the nature of the work environment problem. The results of this study also indicate that never or seldom experiencing fair leadership, role clarity, equality, decision demands and a good social climate is associated with an increased risk of production loss due to work environment problems compared to experiencing these circumstances very often/always or most of the time. It is suggested that future research replicates this study in other working populations to test the transferability of the results.

## References

[CR1] Aronsson G, Gustafsson K (2005). Sickness presenteeism: prevalence, attendance-pressure factors, and an outline of a model for research. J Occup Environ Med.

[CR2] Aronsson G, Gustafsson K, Dallner M (2000). Sick but yet at work. An empirical study of sickness presenteeism. J Epidemiol Community Health.

[CR3] Bakker AB, Demerouti E (2007). The job demands-resources model: state of the art. J Manag Psychol.

[CR4] Bergström G, Jensen I, Fried I, Björklund C, Grahn A, AHA-group (2004) Work and health in the processing and engineering industries (in Swedish). Final report, part one. Department of Clinical Neuroscience. Section for Personal Injury Prevention. Karolinska Institute, Stockholm

[CR5] Bergström G, Björklund C, Fried I (2008). A comprehensive workplace intervention and its outcome with regard to lifestyle, health and sick leave: the AHA study. Work.

[CR6] Brooks A, Hagen SE, Sathyanarayanan S, Schultz AB, Edington DW (2010). Presenteeism: critical issues. J Occup Environ Med.

[CR7] Callen BL, Lindley LC, Niederhauser VP (2013). Health risk factors associated with presenteeism in the workplace. J Occup Environ Med.

[CR8] Dallner M (2000). Validation of the General Nordic Questionnaire (QPSNordic) for psychological and social factors at work.

[CR9] Dew K, Keefe V, Small K (2005). ‘Choosing’ to work when sick: workplace presenteeism. Soc Sci Med.

[CR10] Elwér S, Harryson L, Bolin M, Hammarström A (2013). Patterns of gender equality at workplaces and psychological distress. PLoS One.

[CR11] Hansen CD, Andersen JH (2008). Going ill to work—what personal circumstances, attitudes and work-related factors are associated with sickness presenteeism?. Soc Sci Med.

[CR12] Johns G (2010). Presenteeism in the workplace: a review and research agenda. J Organ Behav.

[CR13] Karlsson ML, Bergström G, Bjorklund C, Hagberg J, Jensen I (2013). Measuring production loss due to health and work environment problems—construct validity and implications. J Occup Environ Med.

[CR14] Lack DM (2011) Presenteeism revisited. Complet Rev AAOHN J 59:77–89; quiz 90-71 10.3928/08910162-20110126-01 21323209

[CR15] Larsson G (2003). A comprehensive system for leader evaluation and development. Leadersh Organ Dev J.

[CR16] Lerner D, Adler DA, Rogers WH (2010). Work performance of employees with depression: the impact of work stressors. Am J Health Promot.

[CR17] Luchman JN, González-Morales MG (2013). Demands, control, and support: a meta-analytic review of work characteristics interrelationships. J Occup Health Psychol.

[CR18] Robertson I, Leach D, Doerner N, Smeed M (2012). Poor health but not absent: prevalence, predictors, and outcomes of presenteeism. J Occup Environ Med.

[CR19] Roos P, Jensen I, Ødegaard F, Bergström G, Bertilson H, Färe R. (2005) Health and productivity (in Swedish). Final report, part 2, aha-study; work and health in the processing and engineering industries. Department of Clinical Neuroscience. Section for personal injury prevention. Karolinska Institute, Institute for applied economics, Malmö, pp 1–16

[CR20] Spector P (2006). Method variance in organizational research: truth or urban legend?. Organ Res Methods.

[CR21] Stewart WF, Ricci JA, Chee E, Morganstein D (2003). Lost productive work time costs from health conditions in the United States: results from the American Productivity Audit. J Occup Environ Med.

[CR22] Tengblad E (2010). Medarbetarskap på 60 minuter.

[CR23] van den Berg TI, Robroek SJ, Plat JF, Koopmanschap MA, Burdorf A (2011). The importance of job control for workers with decreased work ability to remain productive at work. Int Arch Occup Environ Health.

[CR24] Wynne-Jones G, Buck R, Varnava A, Phillips CJ, Main CJ (2011). Impacts on work performance; what matters 6 months on?. Occup Med (Lond).

[CR25] Zou G (2004). A modified poisson regression approach to prospective studies with binary data. Am J Epidemiol.

